# Promising inhibition of diabetes-related enzymes and antioxidant properties of *Ptilostemon casabonae* leaves extract

**DOI:** 10.1080/14756366.2023.2274798

**Published:** 2023-10-31

**Authors:** Cinzia Sanna, Antonella Fais, Benedetta Era, Giovanna L. Delogu, Enrico Sanna, Laura Dazzi, Antonella Rosa, Arianna Marengo, Patrizia Rubiolo, Antonio De Agostini, Sonia Floris, Francesca Pintus

**Affiliations:** a Department of Life and Environmental Sciences, University of Cagliari, Cagliari, Italy; b Department of Biomedical Science, University of Cagliari, Cagliari, Italy; c Department of Drug Science and Technology, University of Turin, Turin, Italy

**Keywords:** α-Glucosidase, α-amylase, diabetes, enzyme inhibition, *Ptilostemon casabonae*

## Abstract

Type 2 diabetes (T2D) is a progressive metabolic disorder of glucose metabolism. One of the therapeutic approaches for the treatment of T2D is reducing postprandial hyperglycaemia through inhibition of the digestive enzymes α-glucosidase and α-amylase. In this context, aimed at identifying natural products endowed with anti-T2D potential, we focused on *Ptilostemon casabonae* (L.) Greuter, a species belonging to Asteraceae family. Enzymatic inhibition, antioxidant activity, phenolic composition and cellular assays were performed. This study revealed that the *P. casabonae* hydroalcoholic extract exerts a potent inhibitory activity against α-glucosidase. This activity is supported by an antioxidant effect, preventing ROS formation in a stressed cellular system. HPLC-PDA-MS/MS analysis, revealed a complex polyphenolic fraction. Among the tested pure compounds, 1,5-dicaffeoylquinic acid, apigenin and rutin displayed good α-glucosidase inhibitory activity. Our study suggested new potential of *P. casabonae* encouraging us to further testing the possible therapeutic potential of this extract.

## Graphical Abstract

**Figure:**
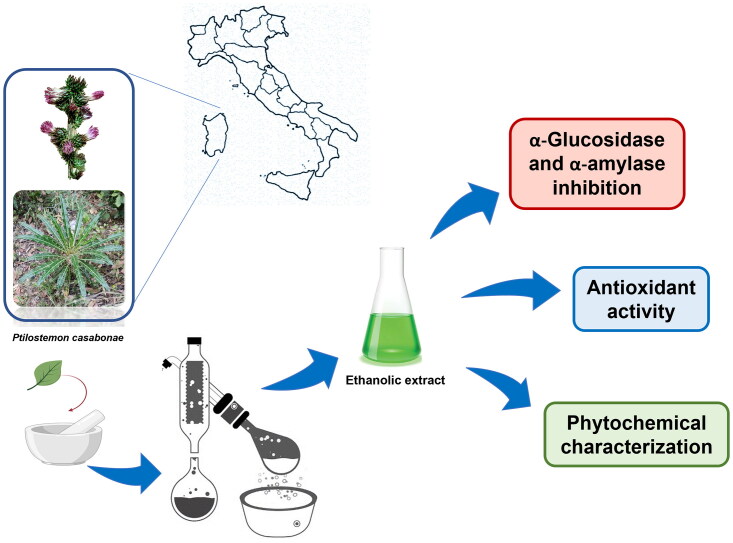

## Introduction

*Ptilostemon casabonae* (L.) Greuter is a wild edible species belonging to the Asteraceae family, subendemic to Sardinia (Italy), Corsica and Hyères Island (France)^1^. It is a spiny herb, up to 150 cm in height, with linear-lanceolate leaves provided with thorns bundles in all the leaf edges and purple inflorescences appearing from May to August. It grows in dry and open habitats, indifferently in calcareous or siliceous soils, between 400 and 1200 m a.s.l. Aerial parts of *P. casabonae* are used in Sardinian traditional medicine as anti-spasmodic^2^.

Even though many Sardinian endemic species have been reported as source of unique and structurally diverse compounds endowed with promising biological activities^3^^,^^4^, the majority of them still remain understudied^5^.

Previous studies focused on the phytochemical analysis of the extracts obtained from its aerial parts, providing information on its primary and specialised metabolites. A remarkable amount of phenolic and amino compounds has been reported^6^^,^^7^, making this plant a good candidate for food supplements and functional foods. However, as far as we know, more information on the biological activities of this species is needed. These considerations encouraged us to investigate its antioxidant and antidiabetic potential.

Diabetes is a progressive metabolic disorder of glucose metabolism. It represents a global public health issue, affecting 537 million adults (20–79 years) worldwide, with this number expected to rise to 783 million by 2045^8^. Moreover, 45% of people live with undiagnosed diabetes, predominantly type 2, so the incidence of the disease should be considered even higher.

Type 1 diabetes is caused by impaired insulin production by pancreatic β-cells and a daily insulin injection is required to keep blood glucose level within an appropriate range. Type 2 diabetes (T2D) is the most prevalent type of diabetes, representing more than 90% of all diabetes worldwide, and it is mainly characterised by insulin resistance or β-cell dysfunction^9^. The causes of T2D are not exactly understood. Still, it is known that the disease onset is closely associated with various risk factors such as overweight and obesity, increasing age, ethnicity, and family history. Strategies for T2D management are oriented towards a correct lifestyle, including a healthynutrition, regular physical activity, quitting smoking, and maintenance of healthy body weight. However, oral medication is needed if this approach is insufficient to control blood glucose level. The treatment can include a single antidiabetic medication, but many combination therapy options are often used. Several drugs with different modes of action are available, including the α-glucosidase inhibitors.

α-Glucosidase (EC 3.2.1.3) and α-amylase (EC 3.2.1.1) enzymes can hydrolyse carbohydrates and therefore represent important therapeutic targets in controlling postprandial hyperglycaemia in diabetic disease^10^. Salivary and pancreatic α-amylases catalyse the endohydrolysis of α-D-1,4-glycosidic bonds in starch, producing maltose and other oligosaccharides, while intestinal α-glucosidase catalyses the hydrolysis of terminal 1,4-linked α-D-glucose residues from nonreducing ends of isomaltose oligosaccharides, yielding free D-glucose, which can be absorbed by intestinal cells. Inhibitors of these enzymes slow down carbohydrate digestion, thus prolonging overall digestion time, causing a reduction in glucose absorption, thereby moderating the postprandial blood glucose elevation. Therefore, a therapeutic approach to treat diabetes is to inhibit these enzymes to decrease postprandial hyperglycaemia.

Moreover, it is well known that there is a strong link between hyperglycaemia, hyperglycaemic-induced oxidative stress and the development and progression of T2D. Oxidative stress stimulates the generation of inflammatory mediators, and inflammation, in turn, improves the production of reactive oxygen species (ROS) that can lead to many diseases, including T2D. ROS accumulation can promote cellular damage and contribute to diabetic complications such as cardiovascular alterations^11^. Therefore, therapies based on the downregulation of ROS generation may be pivotal in treating T2D and controlling diabetic complications.

Thus, in this context, the present study aims to obtain and characterise a novel potent antidiabetic extract from *P. casabonae* with concomitant antioxidant activity.

## Materials and methods

### Chemicals

Formic acid (>98% purity), acetonitrile (LC-MS grade), methanol, rutin, quercetin, apigenin and kaempferol were purchased from Merck (Darmstadt, Germany). Apigenin 7-O-glucoside, quercetin 3-O-glucoside, kaempferol 3-O-rutinoside and luteolin were purchased from Extrasynthese (Genay Cedex, France). Cynarin, chlorogenic acid, 1,5-dicaffeoylquinic acid, 3,5-dicaffeoylquinic acid, 4,5-dicaffeoylquinic acid, luteolin 7-O-glucuronide, apigenin, 7-O-glucoside, luteolin 7-O-glucoside and diosmetin were purchased from Phytolab (Vestenbergsgreuth, Germany). Bond Elut Jr 500 mg SPE-C18 cartridge was from Agilent (Santa Clara, California, USA). A Milli-Q purification system (Millipore, Bedford, MA, USA) was used to obtain the deionised water (18.2 MΩ cm). All chemicals used for biological assays were obtained as pure commercial products from Sigma Chemical Co (St. Louis, USA) and used without further purification. Spectrophotometric determinations were obtained with an Ultrospec 2100 spectrophotometer (Biochrom Ltd, Cambridge, England) using a 1 cm length path cell.

### Plant material

Leaves of *P. casabonae* were collected from Jerzu (Sardinia, Italy) in May 2021. The plant was identified by Cinzia Sanna, and a sample was deposited at the General Herbarium (HerbariumCAG) of the University of Cagliari (Cagliari, Italy), with the voucher specimen (CAG 796/v1). The plant, even though endemic, is not under the protection of national or international regulations; thus, it was not necessary a specific permission for its harvesting. Leaves were dehydrated at 40 °C by using a ventilated stove, and powdered by an electric grinder.

### Extract preparation

Powdered leaves of *P. casabonae* (32 g) were subjected to three consecutive and exhaustive extractions with 80% ethanol (800 ml × 3) for 24 h each. Extracts were then pooled, filtered, and concentrated under vacuum at 40 °C to eliminate the alcohol phase. The remaining aqueous phase was frozen to −80 °C and then lyophilised, yielding 3.7 g of crude extract that was kept at −20 °C until chemical analyses and biological assays. Before use, unless otherwise specified, the extract was dissolved in water at the desired concentration for chemical and biological assays.

### Characterisation of the extract through HPLC-PDA-MS/MS

A Shimadzu Nexera × 2 system coupled with an SPD-M20A photodiode array detector and a Shimadzu LCMS-8040 triple quadrupole system with electrospray ionisation source (Shimadzu, Dusseldorf Germany) was used for the qualitative and quantitative analyses of the extract, as previously reported by Marengo et al.^6^. Analytical conditions are: column Ascentis® Express C18 (15 cm × 2.1 mm, 2.7 μm, Supelco, Bellefonte, USA); mobile phases: A: water (added with 0.1% formic acid), B: acetonitrile (added with 0.1% formic acid); flow rate: 0.4 ml/min; UV spectra: 220–600 nm; column T: 30 °C; Gradient program: 5% B for 5 min, 5–15% B in 17 min, 15–25% B in 10 min, 25–75% B in 12 min, 75–100% B in 10 min, 100% B for 1 min. Total time (pre- and post-run) was 60 min. MS operative conditions: Heat block temperature 200 °C; desolvation line (DL) temperature: 250 °C; nebuliser gas flow rate: 3 L/min; drying gas flow rate: 15 L/min. Mass spectra were acquired in both positive and in negative full-scan modes (range 100–1000* m/z*, event time 0.5 s). Product Ion Scan mode: collision energy: ESI+: − 35.0 V and ESI−: 35.0 V, event time: 0.2 s. Quantification was performed in the UV, with each compound quantified by the external calibration method using the relative commercial standard compound at its maximum absorption (325 nm for chlorogenic and 1,5 dicaffeoylquinic acid; 350 nm for rutin; 340 nm for apigenin). Succinyl dicaffeoylquinic acid, for which no commercial standard was available, was quantified using 1,5-dicaffeoylquinic acid calibration curve. For the standard solutions and the extracts three replicates of analyses were performed.

### SPE-C18 cartridge purification

Succinyl dicaffeoylquinic acid-enriched fraction of *P. casabonae* leaves extract was prepared on an SPE-C18 cartridge. A conditioning of the cartridge was first carried out with 4 ml of methanol and 4 ml of deionised water. 10 mg of the dried extract (DE) was resuspended in 1 ml of water, loaded onto the Bond Elut Jr 500 mg SPE-C18 cartridge, and eluted with 5 ml of deionised water. The fraction was then freeze-dried, yielding approximately 2 mg. This procedure was repeated to obtain a sufficient amount of the fraction for the biological tests. The fraction obtained was analysed by HPLC-PDA-MS/MS for its chemical characterisation and quantification of the major constituents.

### Fatty acid analysis

Aliquots (2 mg, in ethanol solution) of DE were subjected to mild saponification as previously reported^12^. Dried saponifiable fractions, dissolved in acetonitrile, were analysed by high-performance liquid chromatography (HPLC). Fatty acids (FA) analyses were performed after saponification, and carried out with an Agilent Technologies 1100 HPLC (Agilent Technologies, Palo Alto, CA) equipped with a DAD and an Infinity 1260 ELSD detector (HPLC-DAD/ELSD). FA were eluted with CH3CN/H2O/CH3COOH (75/25/0.12, *v/v/v)* as mobile phase at a flow rate of 2.3 ml/min, using a XDB-C18 Eclipse column^12^. Saturated FA (SFA) were detected using ELSD, while unsaturated FA (UFA) at wavelength of 200 nm (DAD). Chromatogram data recording and integration were carried out through an Agilent OpenLAB Chromatography data system. The FA identification was performed using standard compounds and UV spectra (for UFA). FA calibration curves were constructed using the reference standards and were found to be linear (DAD) and quadratic (ELSD) (correlation coefficients >0.995)^12^.

### Determination of the total phenolics and flavonoids

The total phenolic content in the extract was determined by using the method of Folin-Ciocalteu, using gallic acid as a reference standard, while aluminium nitrate assay was used to determine the total flavonoid content^13^. Phenolic and flavonoid concentration was expressed as mg of gallic acid equivalents (GAE) or mg of quercetin equivalents (QE) per g of DE, respectively.

### ABTS radical scavenging activity

2,2′-Azinobis-(3-Ethylbenzothiazoline-6-Sulfonic Acid (ABTS) radical-scavenging activity was determined with the method as previously reported^13^, using Trolox (6-hydroxy-2,5,7,8-tetramethylchromane-2-carboxylic acid) as the standard reference. Briefly, the reaction between the reduced ABTS (7 mM) with potassium persulfate (2.45 mM) in an aqueous solution at room temperature for 24 h, was performed to produce free radical ABTS^.+^. Then, different concentrations of extract (10 μL) were added to 1 ml of ABTS^.+^, and the absorbance at 734 nm was recorded after 1 min. The activity was expressed as the concentration of sample necessary to give a 50% reduction in the original absorbance (EC_50_).

### FRAP assay

Ferric reducing antioxidant power assay (FRAP) was conducted as previously described^14^, with slight modifications. An amount of 10 µL of the extract was mixed with 2 ml of the TPTZ–ferric solution and than incubated for 4 min at room temperature in the dark. The absorbance was read at 593 nm and the result was expressed as ferrous equivalents (FE; mg Fe^2+^ equivalents/mL solution). Ascorbic acid was used as reference compound.

### Enzyme inhibition

The results of the catalytic activities were reported as the percentage of the blank control. The concentrations of extract required to give 50% inhibition of enzyme activity (IC_50_) were determined. Acarbose was used as a reference compound for both enzymes. The Lineweaver-Burk double reciprocal plot was used to determine the mode of α-glucosidase inhibition by performing assays at different concentrations of substrate and extract or compound. Grafit 7 (Erithacus, East Grinstead, UK) was used as data analysis software.

#### α-Glucosidase inhibition assay

α-Glucosidase inhibitory activity was analysed following the method described by Delogu et al.^15^. The reaction mixture consisted of 120 μL of 0.1 M sodium phosphate buffer (pH 6.8), 40 μL of *Saccharomyces cerevisiae* α-glucosidase (0.125 U/mL) and 20 μL extract or single compound at different concentrations. It was first incubated for 15 min at 37 °C. Then, 20 µL of p-nitrophenyl α-D-glucopyranoside solution (pNPG, 5 mM) was added. After further 15 min of incubation at 37 °C, the reaction was stopped by adding 50 μL of 0.2 M sodium carbonate solution. α-Glucosidase activity was determined with a 96-well microplate reader by measuring the absorbance at 405 nm. The mode of inhibition was determined, and the equilibrium constant for binding with the enzyme–substrate complex (K_IS_) was obtained from the vertical intercepts plotted versus inhibitor concentration.

#### α-Amylase inhibition assay

The reaction mix used to determine the α-amylase activity consisted of 60 μL of sodium phosphate buffer (50 mM, pH 7.0), 20 μL of NaCl (1 M) and 40 μL of α-amylase from porcine pancreas (1 mg/mL). The solution was incubated at 37 °C for 10 min in the absence or presence of extract or a single compound. After incubation, 80 μL of the 2-chloro-4-nitrophenyl-α-D-maltotrioside substrate solution (CNPG3, 2.5 mM) were added, and the amount of 2-chloro-nitrophenol released by the enzymatic hydrolysis was consequently monitored at 405 nm^15^.

### Cell culture and intracellular ROS levels

Caco-2 cells (American Type Culture Collection ATCC, Manassas, VA, USA) were cultured at 37 °C in a humidified atmosphere with 5% CO_2_ in Dulbecco’s Modified Eagle Medium (DMEM) containing 10% foetal bovine serum (FBS; Gibco, NY, USA), and 1% penicillin/streptomycin. As previously described^14^, cell viability was measured by using 3–(4,5-dimethylthiazol-2-yl)-2,5-diphenyltetrazolium bromide (MTT) reagent after seeding 104 cells per well into a 96-well plate and incubating for 24 h with different concentrations of *P. casabonae* extract (0–100 μg/mL).

Caco-2 cells were also treated with the same concentrations of extract to evaluate the cellular ROS levels by using the 2′,7′-dichlorofluorescein diacetate (DCFH-DA) method. After 24 h, cells were incubated with DCFH-DA (10 μM) at 37 °C for 30 min and then treated with 1 mM H_2_O_2_. The fluorescence intensity of DCF was immediately measured with a fluorescent plate reader at the excitation wavelength of 485 nm and emission wavelength of 530 nm. Readings were taken every 5 min for 60 min^14^.

### Statistical analysis

All assays were conducted in triplicates and the data were expressed as mean ± standard deviation (SD). GraphPad Prism software version 9 (San Diego, CA, USA) was used to evaluate statistical differences. Comparison between groups was performed by one-way analysis of variance (ANOVA) followed by the Tukey Multiple Comparisons Test. A *p* values of less than 0.05 was considered statistically significant.

## Results and discussion

### Total polyphenols, total flavonoids content and antioxidant activity

Polyphenols and flavonoids are important bioactive compounds endowed with strong antioxidant properties^16^^,^^17^. In this work, we first evaluated the total phenolic and flavonoid content of a hydroalcoholic extract obtained from *P. casabonae* leaves. Then, we investigated its radical scavenging activities by the ABTS assay. The results obtained, shown in Table 1, revealed a rich phenolic fraction since the total phenolic content was found to be 321.31 ± 4.06 mg GAE/g of DE, and the flavonoid content was found 214.40 ± 3.67 mg QE/g of DE. Regarding the antioxidant activity, the extract displayed an EC_50_ value of 10.46 ± 0.26 µg/mL, close to that of Trolox, used as a standard. This result was also confirmed by FRAP assay that showed a strong reducing power of *P. casabonae* extract.

**Table 1.** Evaluation of antioxidant activity, total phenolic and flavonoid content of *P. casabonae* leaves extract.

Sample	ABTS	FRAP	Total Phenolic	Total Flavonoid
	EC_50_ µg/mL	mg FE/mL	mg GAE/g DE	mg QE/g DE
*P. casabonae* extract	10.46 ± 0.26	2.04 ± 0.11	321.31 ± 4.06	214.40 ± 3.67
Trolox	3.4 ± 0.3			
Ascorbic acid		1.81 ± 0.13		

### Fatty acid composition

Since unsaturated fatty acids are described as competitive inhibitors of starch digestive enzymes (α-amylase and α-glucosidase)^18^, the fatty acid composition of the extract was performed.

**Figure 1.**
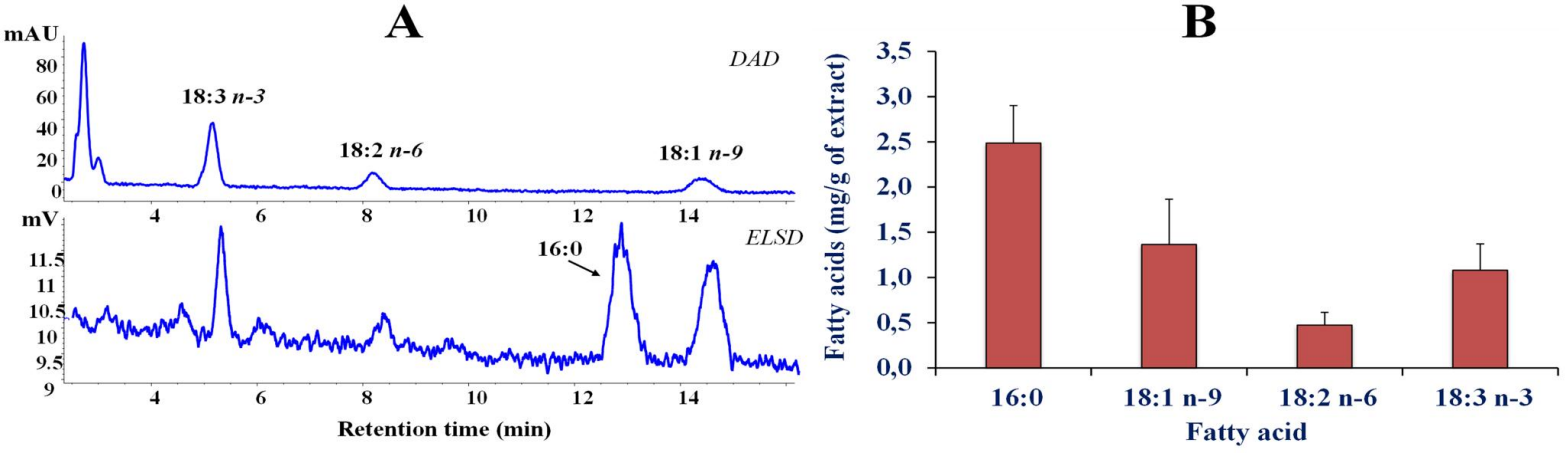
Fatty acid (FA) chromatographic profiles (HPLC analysis) obtained by DAD (200 nm, unsaturated FA) and ELSD (saturated FA) detection (A) and FA composition (expressed as mg/g of extract) determined by HPLC-DAD/ELSD analysis (B) of *P. casabonae* leaf extract after saponification. Analysis was performed in quadruplicate and all data are expressed as mean values ± standard deviations (SD) (*n* = 4).

Figure 1 shows the representative chromatographic profile of the main fatty acids (FA) in the leaves extract of *P. casabonae* (Figure 1(A)) obtained by HPLC-DAD/ELSD analysis^12^. The combined use of the two detectors allowed to quantify both saturated (SFA, ELSD detection) and unsaturated (UFA, UV detection) FA in one single analysis^12^. The most represented FA of the extract were palmitic acid (16:0, 2.49 ± 0.42 mg/g of dry weight), oleic acid (18:1 n-9, 1.36 ± 0.5 mg/g of dry weight), linolenic acid (18:3 n-3, 1.08 ± 0.29 mg/g of dry weight), and linoleic acid (18:2 n-6, 0.47 ± 0.14 mg/g of DE) (Figure 1(B)). The amount of lipids in the hydroalcoholic leaves extract of *P. casabonae* was very low, and the total FA content approximately accounted for 0.54% of the extract.

The FA profile of *P. casabonae* leaves extract was different from that reported for the fruits of *Ptilostemon afer* (Jacq.) Greuter *subsp. eburneus* Greuter, characterised by a high content (expressed as %, DE) of linoleic acid (58.5 ± 0.1), followed by oleic acid (19.2 ± 0.1), and palmitic acid (9.0 ± 0.1)^19^. The *P. casabonae* FA profile was similar to those previously reported for lipid extracts obtained from the leaves of various medicinal and edible plants^20^, characterised by palmitic acid as the dominant FA, followed by oleic, linolenic, and linoleic acids in different proportions.

Our results on lipid content add useful information to the chemical characterisation of this understudied subendemic plant, including its primary metabolites. These compounds, together with specialised metabolites, are very important for their nutritional and healthy properties, especially considering that this species, as well as the other wild thistles, are traditionally used for food. As far as we know, this is the first report on lipid content of *P. casabonae* leaves, while a previous article investigated the content of amino compounds, revealing proline, asparagine, glutamine and glutamic acid as the main constituents in the acidified hydroalcoholic extract of the aerial parts of this species^7^.

### α-Glucosidase and α-amylase inhibition of P. casabonae leaves extract

Table 2 reports the ability of the extract of *P. casabonae* leaves to reduce α-amylase and α-glucosidase activities. The extract weakly inhibits α-amylase, while it exhibits potent inhibitory activity against α-glucosidase (Table 2). Indeed, *P. casabonae* extract was found to be about 7 times more active (*p* < 0.001) than the standard inhibitor, acarbose, with an IC_50_ value of 12.20 ± 0.99 µg/mL. Acarbose is commonly used to treat hyperglycaemia in T2D patients; however, the use of this drug is accompanied by several side effects, probably due to a greater inhibition of α-amylase in comparison to α-glucosidase inhibition^21^. *P. casabonae* extract instead showed a better inhibition against α-glucosidase, making this extract a good candidate for further and more detailed studies.

**Table 2.** Inhibitory activity of *P. casabonae* extract against α-glucosidase and α-amylase.

Sample	α-Glucosidase		α-Amylase	
	Inhibition %	IC_50_	Inhibition %	IC_50_
	(100 µg/mL)	(µg/mL)	(100 µg/mL)	(µg/mL)
*P. casabonae* extract	94.2 ± 5.2	12.20 ± 1.0	38.9 ± 1.7	107.0 ± 4.1
Acarbose		90 ± 7.3		8.0 ± 0.7

**Figure 2.**
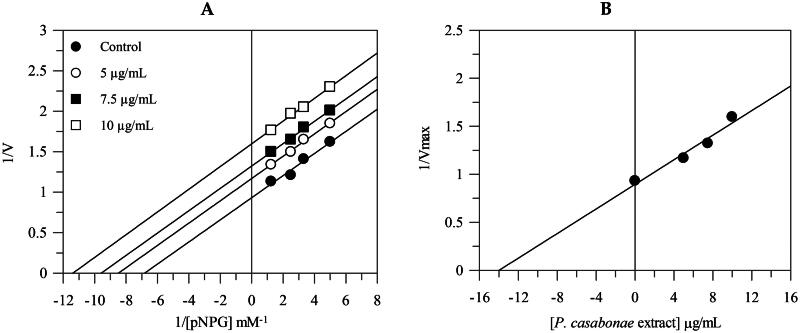
Inhibition of α-glucosidase activity. Lineweaver–Burk analysis (A) and secondary plot (B) using different concentrations of the extract.

The Lineweaver-Burk plots showed that the extract behaves with an uncompetitive mode of inhibition since the kinetic analysis of the extract gives a family of parallel lines for increasing extract concentrations (Figure 2). The equilibrium constant for binding with the enzyme-substrate complex (K_IS_) was calculated from the replotting of the intercepts (1/*V*_max_) versus the inhibitor concentration, resulting in a value of 13.9 μg/mL.

### Cell viability and intracellular ROS level

Since the extract of *P. casabonae* exhibited a good antioxidant activity on ABTS assay, we decided to investigate whether it exerts antioxidant effects also by inhibiting H_2_O_2_-induced ROS generation in a cellular model.

**Figure 3.**
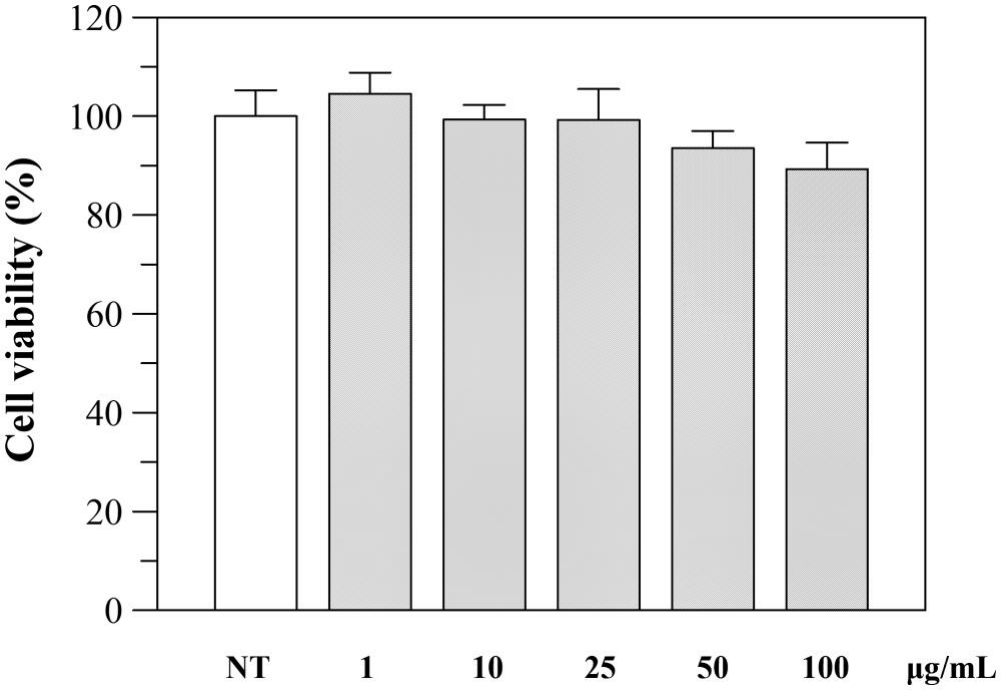
Effect of *P. casabonae* extract on Caco-2 cell viability after 24 h incubation with the extract at different concentrations. No statistically differences between treated and non-treated (NT) cells were observed.

The effect of the extract was first investigated on cell viability using Caco-2 as cell line. Caco-2 cells were exposed for 24 h to various concentrations of the extract and then examined with the MTT test. As shown in Figure 3, the extract was not cytotoxic, and only a little decrease (viability of 85%) was observed at 100 μg/mL.

**Figure 4.**
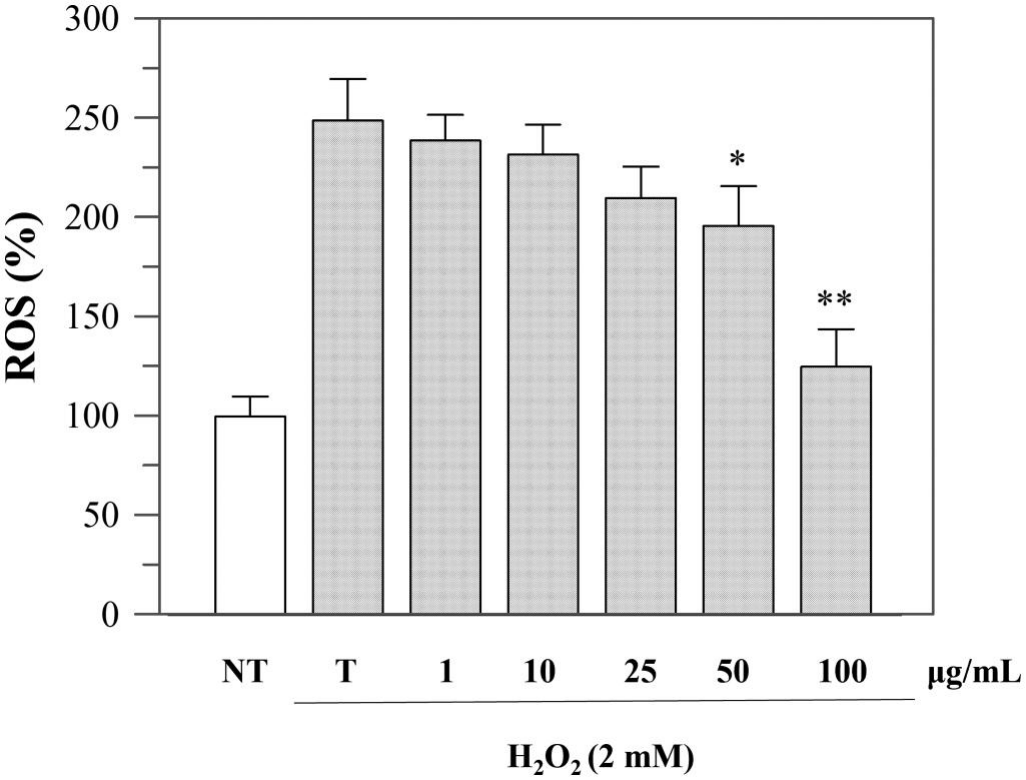
Inhibition of H_2_O_2_-induced ROS generation (1 h-incubation with 2 mM H_2_O_2_) by *P. casabonae* extract on Caco-2 cells. NT, non-treated cells; T, cells treated with H_2_O_2_ only. Asterisks indicate values statistically different (*p < 0.05; ** *p* < 0.0001) from cells treated with H_2_O_2_ only (T). All H_2_O_2_-treated samples were statistically different from NT with the exceptions of 100 μg/mL *P. casabonae* extract.

Since the viability was not affected up to a concentration of 100 μg/mL, we performed further cellular experiments using up to this extract concentration. To investigate the protective effects against oxidative stress, we evaluated the intracellular ROS levels before and after H_2_O_2_-induced oxidative stress and after incubation with the extract. The experiment was performed using DCFHDA, which is hydrolysed by the endogenous esterases to DCFH. The oxidation of DCFH to DCF, induced by H_2_O_2_, was quantified through a spectrophotometer. As shown in Figure 4, the incubation with H_2_O_2_ significantly increased ROS levels in Caco-2 cells, but treatment with *P. casabonae* extract inhibited H_2_O_2_-induced ROS generation in a dose-dependent manner. These results confirm what observed in the antioxidant assays, and suggest a potential role of *P. casabonae* extract in reducing ROS formation in cells. At the concentration of 100 μg/mL, ROS formation was reduced back to non-treated cells level, since no statistically differences were observed between the two samples. At the same concentration, *P. casabonae* extract exerts almost totally inhibition against α-glucosidase activity, with a concomitant effect against α-amylase (Table 2), also showing no cytotoxic effect (Figure 3).

### Phytochemical study of P. casabonae extract

Based on the interesting antioxidant and potential antidiabetic properties observed, the characterisation of the extract of *P. casabonae* leaves was carried out by HPLC-PDA-MS/MS to identify the main compounds which could be responsible for the observed activity. The phytochemical analysis, in agreement with the total phenolic and flavonoid content results described above, revealed a complex polyphenolic fraction. Our results are in line with a previous study in which caffeoylquinic acid derivatives are described as the most representative group of specialised metabolites detected in this species^6^.

**Figure 5.**
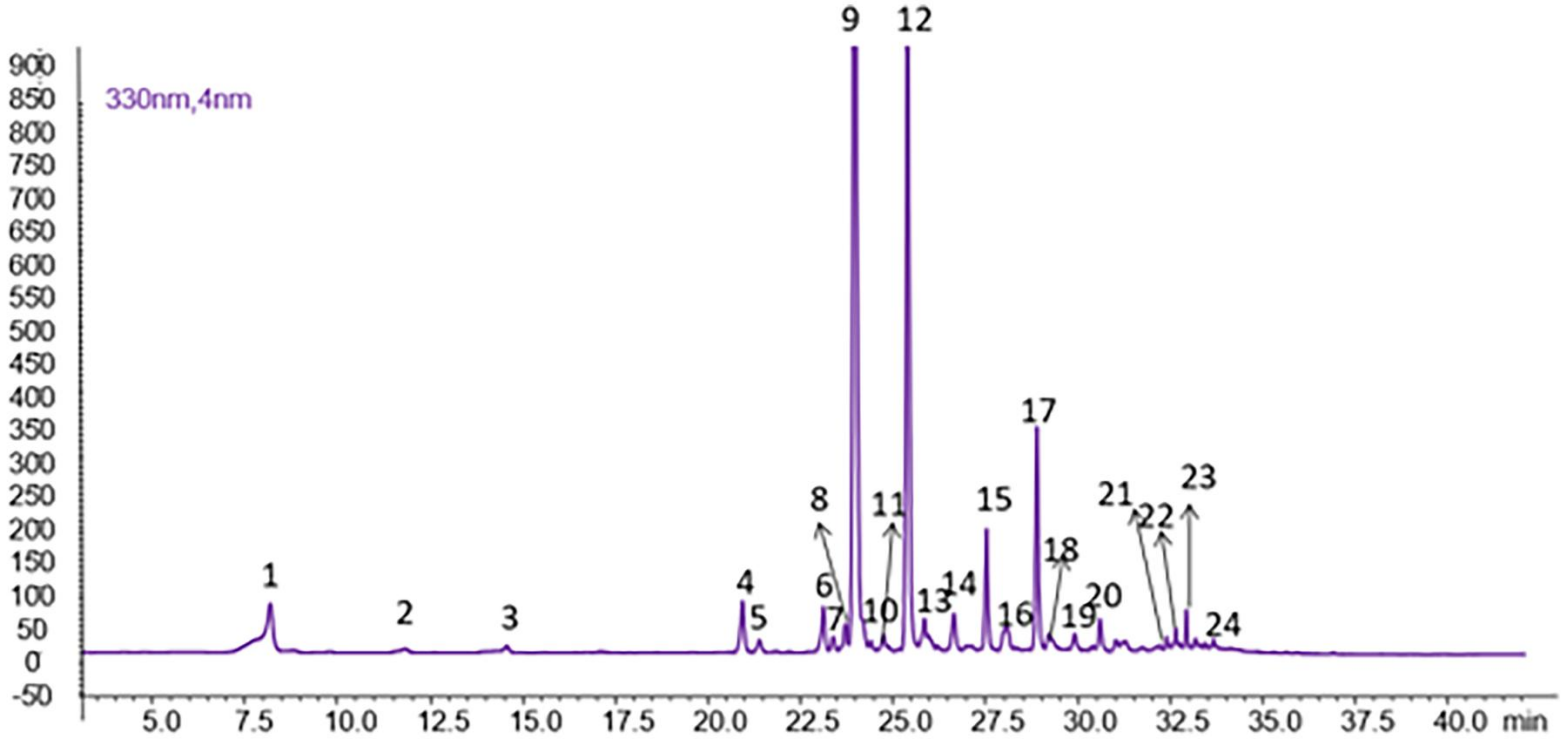
Chromatographic profile of *P. casabonae* leaves extract (*λ* = 330 nm). Compound numbers refer to Table 3.

As shown in Figure 5, twenty-four peaks were detected, including quercetin, luteolin, kaempferol, apigenin and diosmetin *O*-glycosides, in addition to the caffeoylquinic acid derivatives.

The list of all compounds detected in the extract is reported in Table 3. Among these, fourteen were confirmed by the coinjection of commercial standards. For compounds for which no reference standards were available, the putative identification was performed by comparison with literature data, considering their maximum UV absorbance, the molecular weight based on the pseudomolecular ions detected in the positive and negative ioniza-tion modes and the characteristic fragments obtained by tandem mass spectrometry.

**Table 3.** List of identified and tentatively identified compounds in *P. casabonae* leaves extract.

N°	RT	*λ*_max_ (nm)	ESI +	ESI−	MW g/mol	PIS+	PIS−	Compound name^a^	References
1	8.196	325	355	353	354	163	191	5-*O*-Caffeoylquinic acid (Chlorogenic acid)^b^	^6^
2	11.850	311	339	337	338	119,147	119,145,163,173,191	Coumaroylquinic acid^c^	^22^ ^,^ ^23^
3	14.598	321/299	517	515	516	163	191	1,3-Dicaffeoylquinic acid^1^	^6^
4	20.970	351/255	611	609	610	303	301	Quercetin 3-*O*-rutinoside (Rutin)^b^	^6^
5	21.433	254/349	465	463	464	303	301	Quercetin 3-*O*-glucoside^1^	^6^
6	23.158	326	517	515	516	163	191	Dicaffeoylquinic acid^c^	^6^
7	23.427	254/351	551	549	550	303	301	Quercetin *O*-malonylhexoside^c^	^6^
8	23.754	333/265	595	59	594	287	285	Kaempferol 3-*O*-rutinoside^b^	^6^
9	24.002	327	517	515	516	163	191	1,5-Dicaffeoylquinic acid^b^	^6^
10	24.444	343	625/517	623/515	624/516	625➔317	623➔315	Isorhamnetin -*O*-glucoside -rhamnoside^c^ +	^6^ ^,^ ^24^
						517➔163	515➔191	3,5-Dicaffeoylquinic acid^b^	
11	24.775	251/361	433/465	431/463	432/464	433➔271	431➔269	Apigenin 7-*O*-glucoside^b^ + Quercetin *O*-hexoside isomer^c^	^6^
						465➔303	463➔301		
12	25.425	328	617	615	616	163	191	Succinyl-dicaffeoylquinic acid isomer^c^	^6^
13	25.900	329	617/517	615/515	616/516	617➔163	615➔191	Succinyl-dicaffeoylquinic acid isomer^c^ + 4,5-dicaffeoylquinic acid^b^	^6^
						517➔163	515➔191		
14	26.693	319	465/501	463/499	464/500	465➔303	463➔301	*p*-Coumaroyl-caffeoylquinic acid/caffeoyl-*p*-coumaroylquinic acid^2^ + Quercetin *O*-hexoside isomer^c^	^6^
						501➔163,145,135,119	499➔337,191,163		
15	27.572	328	617	615	616	163	191	Succinyl-dicaffeoylquinic acid isomer^c^	^6^
16	28.115	318	601	599	600	163,147	337,191,173	Succinyl-*p*-coumaroyl-caffeoylquinic acid/Succinyl caffeoyl-*p*-coumaroylquinic acid^c^	^6^
17	28.933	330	717	715	716	163	191	Succinyl-succinyl-dicaffeoylquinic acid^c^	^6^
18	29.251	313	485	483	484	119/147	119/145/163/191	di-*O-p*-coumaroylquinic acid^c^	^22^
19	29.953	253/368	303/287	301/285	302/286	287➔135,137,153,161		Quercetin^b^+ luteolin^b^	^6^
						303➔127,137,153,165			
20	30.637	313	585	583	584	119/147	163/191/337	Succinyl di-*O-p*-coumaroylquinic acid isomer^c^	^22^
21	32.444	313	585	583	584	119/147	117/173/191/337	Succinyl di-*O-p*-coumaroylquinic acid isomer^c^	^22^
22	32.684	315	697	695	696	147/303	285/300/365/487/651	Unknown^d^	
23	32.964	317	271	269	270	119,153,163		Apigenin^b^	^6^
24	33.473	314	287/301	285/299	286/300	287➔ 121,137,153,165		Kaempferol^b^ + Diosmetin^b^	^6^
						301➔ 258, 286			

Each peak is described by its relative retention time, UV absorption maximum, pseudomolecular ions, molecular weight (MW), fragments obtained by Product Ion Scan mode (PIS) and compound name. References are also included.

^a^Identification confidence value: Level a: Identified compound through the co-injection of an authentic reference standard; Level b: Hypothesise compound (similarity of chromatographic and spectral data to published data); Level c: Unknown.

All compounds, except for coumaroylquinic acid (**2**), isorhamnetin-*O*-glucoside-rhamnoside (**10**), quercetin *O*-hexoside (**14**), di-*O*-p-coumaroyl quinic acid (**18**), and succinyl di-p-coumaroylquinic acid isomers (**20** and **21**) have been previously found in this species^6^. The phytochemical profile of *P. casabonae* was also found to be similar to that described for other wild thistles^14^^,^^25^^,^^26^. The most representative specialised metabolites belong to the class of hydroxycinnamates, with succinyl-dicaffeoylquinic acid (**12**) and 1,5-dicaffeoylquinic acid (**9**) amounting to 99.82 ± 3.23 and 98.64 ± 1.50 mg/g of DE, respectively. These compounds, together with other flavonoids and phenolic acids (chlorogenic acid (**1**) 21.94 ± 0.12 mg/g of DE, rutin (**4**) 15.65 ± 0.68 mg/g of DE and apigenin (**23**) 0.15 ± 0.006 mg/g of DE) have been tested to verify their role in the activity observed. For all the above-mentioned compounds, authentic commercial standards were used for the biological assays. An exception was made for succinyl-dicaffeoylquinic acid (**12**) for which an enriched fraction was prepared as this standard is not commercially available.

**Figure 6.**
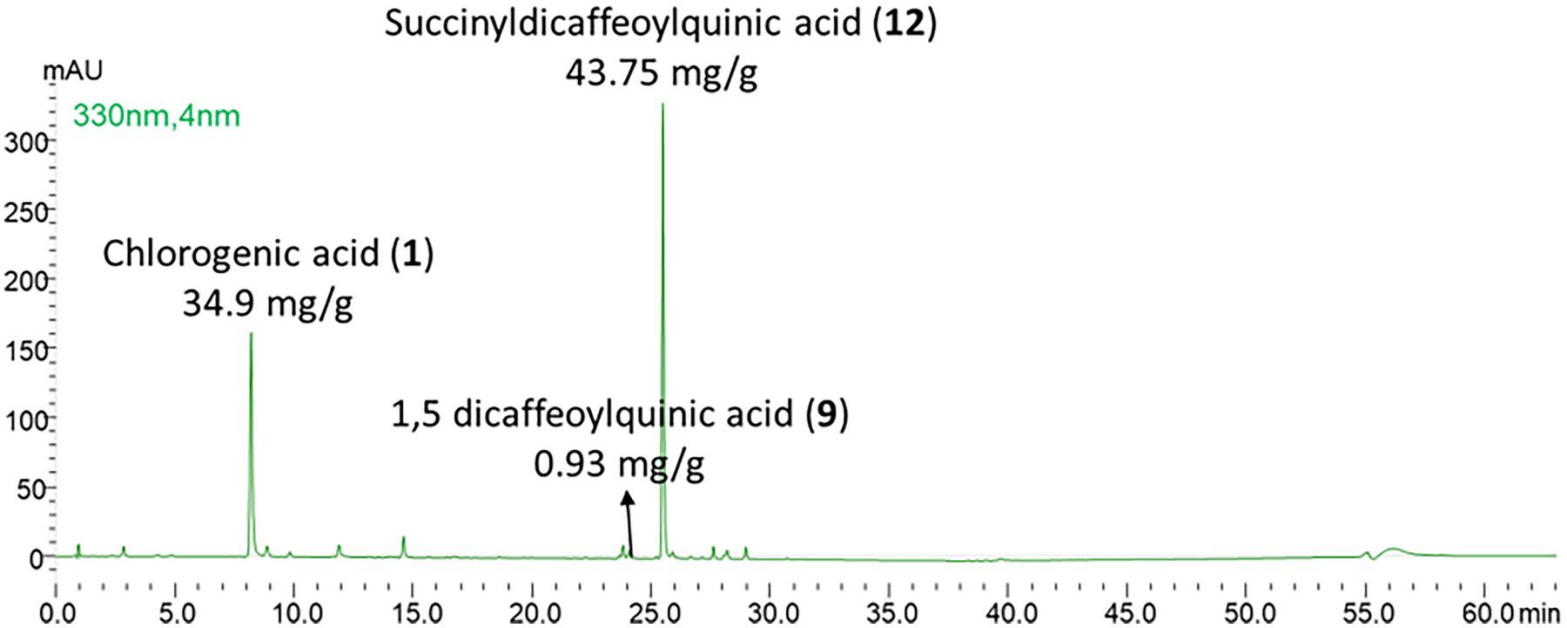
Chromatographic profile of the succinyl-dicaffeoylquinic enriched fraction (*λ* = 330 nm).

As shown in Figure 6, this fraction was composed of succinyl-dicaffeoylquinic acid (43.75 mg/g of fraction), chlorogenic acid (34.9 mg/g of fraction), and 1.5 dicaffeoylquinic acid (in traces). The fractionation carried out did not allow a complete purification of succinyl-dicaffeoylquinic acid due to the chemical similarity of the components characteristic of the extract.

### α-Glucosidase inhibition of P. casabonae compounds

The inhibitory effect of the single compounds as well as the fraction enriched in succinyl-dicaffeoylquinic acid against α-glucosidase activity was evaluated (Table 4).

**Table 4.** IC_50_ value (μg/mL) and inhibition type of *P. casabonae* fraction and single compounds.

Compound	α-Glucosidase	
	IC_50_ (µg/mL)	Mode of inhibition
Fraction	>100	/
1,5-Dicaffeoylquinic acid (**9**)	36.64 ± 0.07	Uncompetitive
Chlorogenic acid (**1**)	>100	/
Apigenin (**23**)	3.88 ± 1.48	Mixed
Rutin (**4**)	20.54 ± 4.45	Mixed

The results show that *P. casabonae* fraction enriched in succinyl-dicaffeoylquinic acid was inactive towards α-glucosidase. This fraction mainly consists of succinyl-dicaffeoylquinic acid and chlorogenic acid and a tiny amount of 1,5-dicaffeoylquinic acid.

Considering that chlorogenic acid has no inhibitory activity against α-glucosidase (Table 4) and the amount of 1,5-dicaffeoylquinic acid present in the fraction is not sufficient to exert an enzyme inhibition, it can also be affirmed that succinyl-dicaffeoylquinic acid does not possess inhibitory potential towards α-glucosidase. Our results showed that 1,5-dicaffeoylquinic acid, apigenin and rutin displayed good inhibitory activity, with IC_50_ values ranging between 3.88 ± 1.48 µg/mL and 36.64 ± 0.07 µg/mL, all significantly lower (*p* < 0.001) than the standard inhibitor acarbose (Table 4).

1,5-Dicaffeoylquinic acid is a compound present in some edible plants (artichoke and sunflower sprouts). Other caffeoylquinic acids detected in our extract, such as 3,5-dicaffeoylquinic acid and 4,5-dicaffeoylquinic acid have been previously found able to inhibit this enzyme^27^, suggesting that the significant inhibition of the *P. casabonae* extract against α-glucosidase enzyme, observed in our study, could be attributable to a synergistic effect between several compounds. The inhibitory activity exhibited by apigenin and rutin support this hypothesis.

**Figure 7.**
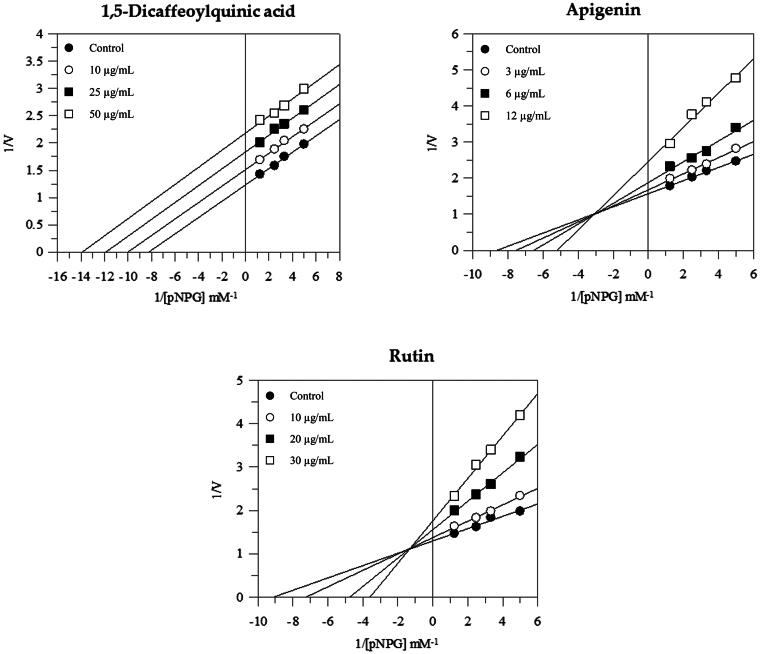
Inhibition of α-glucosidase activity by single compounds. Lineweaver-Burk plot for inhibition of *α*-glucosidase at different compounds concentrations.

The mode of inhibition of the active compounds was also performed and revealed that 1,5-dicaffeoylquinic acid acts as an uncompetitive inhibitor; in fact, the increased concentration of the compound gave rise to a family of parallel straight lines (Figure 7). The constant for binding with enzyme-substrate complex (K_IS_) was calculated from 1/*V*_max_ versus inhibitor concentration and resulted in a value of 69.4 µg/mL (Figure S1).

Instead, both apigenin and rutin exerted a mixed-type inhibition since increasing the concentration of compounds resulted in a family of straight lines with different slopes and y-intercepts, which intersected in the second quadrant. This kinetic analysis indicates that they can bind not only with the free enzyme but also with the enzyme-substrate complex (Figure 7). The equilibrium constants of rutin and apigenin for binding with the enzyme-substrate complex (K_IS_) were found to be 80.2 µg/mL and 19.6 µg/mL respectively, while the equilibrium constants for binding with the free enzyme (K_I_) were obtained from the slope (Km/Vmax) versus inhibitor concentration and resulted in a value of 9.3 µg/mL and 6.5 µg/mL respectively (Figure S1).

## Conclusions

Natural plant products offer unlimited potential for new drugs because their chemical diversity could result in a range of biological activities*. P. casabonae* was revealed to possess a complex polyphenolic composition mainly characterised by caffeoylquinic acid derivatives, followed by flavonoids. Polyphenols and flavonoids are considered important bioactive compounds with strong antioxidant properties. Therefore, radical scavenging activities of *P. casabonae* leaves were investigated, and the result showed good antioxidant activity, also confirmed by the reduction of ROS formation performed on cellular experiments.

It is well known that the production of free radicals and ROS play a significant role in the development of oxidative stress, which is believed to be related to increasing hyperglycaemia, which is associated with T2D. In this respect, *P. casabonae* exhibited potent inhibitory activity against α-glucosidase, being even better than the standard inhibitor, along with a good antioxidant activity. These results suggest that *P. casabonae* could be a promising candidate to provide natural bioactive compounds for treating T2D. Encouraged by the results obtained, further experiments are ongoing in order to evaluate the in vivo effect and develop a nanoformulation delivery system of this extract with the aim of exploiting and possibly enhancing its bioactivity for application in T2D therapy.

## Supplementary Material

Supplemental Material

## References

[1] Fois M, Farris E, Calvia G, Campus G, Fenu G, Porceddu M, Bacchetta G. The endemic vascular flora of sardinia: a dynamic checklist with an overview of biogeography and conservation status. Plants. 2022;11(5):601. 35270071 10.3390/plants11050601 PMC8912449

[2] Atzei AD. Le piante nella tradizione popolare della Sardegna. Sassari: Carlo Delfino Ed; 2003.

[3] Guzzo F, Russo R, Sanna C, Celaj O, Caredda A, Corona A, Tramontano E, Fiorentino A, Esposito F, D'Abrosca B. Chemical characterization and anti-HIV-1 activity assessment of iridoids and flavonols from *Scrophularia trifoliata*. Molecules. 2021;26(16):4777. 34443358 10.3390/molecules26164777 PMC8398805

[4] Sanna C, Scognamiglio M, Fiorentino A, Corona A, Graziani V, Caredda A, Cortis P, Montisci M, Ceresola ER, Canducci F, et al. Prenylated phloroglucinols from *Hypericum scruglii*, an endemic species of Sardinia (Italy), as new dual HIV-1 inhibitors effective on HIV-1 replication. PLOS One. 2018; 13(3):e0195168. 29601601 10.1371/journal.pone.0195168 PMC5877874

[5] Sanna C, Maxia A, Fenu G, Loi MC. So uncommon and so singular, but underexplored: an updated overview on ethnobotanical uses, biological properties and phytoconstituents of Sardinian endemic plants. Plants. 2020;9(8):958. 32751394 10.3390/plants9080958 PMC7465485

[6] Marengo A, Maxia A, Sanna C, Mandrone M, Bertea CM, Bicchi C, Sgorbini B, Cagliero C, Rubiolo P. Intra-specific variation in the little-known Mediterranean plant *Ptilostemon casabonae* (L.) Greuter analysed through phytochemical and biomolecular markers. Phytochemistry. 2019;161:21–27. 30798201 10.1016/j.phytochem.2019.02.005

[7] Marengo A, Maciel LS, Cagliero C, Rubiolo P, Herodes K. Free amino acids and biogenic amines profiling and variation in wild and sub-endemic Cardueae species from Sardinia and Corse. Plants. 2023;12(2):319. 36679032 10.3390/plants12020319 PMC9864185

[8] Magliano DJ, Boyko EJ. IDF diabetes atlas 10th edition scientific committee. 10th ed. Brussels: International Diabetes Federation; 2021. http://www.ncbi.nlm.nih.gov/books/NBK581934/. 35914061

[9] Dirir AM, Daou M, Yousef AF, Yousef LF. A review of alpha-glucosidase inhibitors from plants as potential candidates for the treatment of type-2 diabetes. Phytochem Rev. 2022;21(4):1049–1079. 34421444 10.1007/s11101-021-09773-1 PMC8364835

[10] Floris S, Fais A, Medda R, Pintus F, Piras A, Kumar A, Kuś PM, Westermark GT, Era B. *Washingtonia filifera* seed extracts inhibit the islet amyloid polypeptide fibrils formations and α-amylase and α-glucosidase activity. J Enzyme Inhib Med Chem. 2021;36(1):517–524. 33494628 10.1080/14756366.2021.1874945 PMC7850368

[11] Volpe CMO, Villar-Delfino PH, Dos Anjos PMF, Nogueira-Machado JA. Cellular death, reactive oxygen species (ROS) and diabetic complications. Cell Death Dis. 2018;9(2):119. 29371661 10.1038/s41419-017-0135-z PMC5833737

[12] Rosa A, Maxia A, Putzu D, Atzeri A, Era B, Fais A, Sanna C, Piras A. Chemical composition of *Lycium europaeum* fruit oil obtained by supercritical CO_2_ extraction and evaluation of its antioxidant activity, cytotoxicity and cell absorption. Food Chem. 2017;230:82–90. 28407975 10.1016/j.foodchem.2017.03.019

[13] Floris S, Fais A, Rosa A, Piras A, Marzouki H, Medda R, González-Paramás AM, Kumar A, Santos-Buelga C, Era B. Phytochemical composition and the cholinesterase and xanthine oxidase inhibitory properties of seed extracts from the *Washingtonia filifera* palm fruit. RSC Adv. 2019;9(37):21278–21287. 35521327 10.1039/c9ra02928a PMC9066185

[14] Caddeo C, Tuberoso CIG, Floris S, Masala V, Sanna C, Pintus F. A Nanotechnological approach to exploit and enhance the bioactivity of an extract from *Onopordum illyricum* L. leaves. Plants. 2023; 12(7):1453. 37050078 10.3390/plants12071453 PMC10096861

[15] Delogu GL, Era B, Floris S, Medda R, Sogos V, Pintus F, Gatto G, Kumar A, Westermark GT, Fais A. A new biological prospective for the 2-phenylbenzofurans as inhibitors of α-glucosidase and of the islet amyloid polypeptide formation. Int J Biol Macromol. 2021;169:428–435. 33347933 10.1016/j.ijbiomac.2020.12.117

[16] Brglez Mojzer E, Knez Hrnčič M, Škerget M, Knez Ž, Bren U. Polyphenols: extraction methods, antioxidative action, bioavailability and anticarcinogenic effects. Molecules. 2016;21(7):901. 27409600 10.3390/molecules21070901 PMC6273793

[17] Shen N, Wang T, Gan Q, Liu S, Wang L, Jin B. Plant flavonoids: classification, distribution, biosynthesis, and antioxidant activity. Food Chem. 2022;383:132531. 35413752 10.1016/j.foodchem.2022.132531

[18] Li X, Bai Y, Jin Z, Svensson B. Food-derived non-phenolic α-amylase and α-glucosidase inhibitors for controlling starch digestion rate and guiding diabetes-friendly recipes. LWT. 2022;153:112455.

[19] Kurt A, Ozcan M, Colak N, Ozogul Y, Glew R, Ozogul F, Ayaz FA. Fatty acids of oil and antioxidant capacity of phenolics from fruits of 11 Cardueae (Carduoideae, Asteraceae) taxa from northeast Anatolia (Turkey). Bot Serb. 2019; 43(1):31–45.

[20] Keser S, Celik S, Turkoglu S, Yilmaz Ö, Turkoglu I. Vitamin, sterol and fatty acid contents of some edible and medicinal plants from East and Southeast Anatolia (Turkey). Turk J Pharm Sci. 2015;12(2):46–59.

[21] Oboh G, Ogunsuyi OB, Ogunbadejo MD, Adefegha SA. Influence of gallic acid on α-amylase and α-glucosidase inhibitory properties of acarbose. J Food Drug Anal. 2016;24(3):627–634. 28911570 10.1016/j.jfda.2016.03.003 PMC9336674

[22] Clifford MN, Marks S, Knight S, Kuhnert N. Characterization by LC-MS(n) of four new classes of p-coumaric acid-containing diacyl chlorogenic acids in green coffee beans. J Agric Food Chem. 2006; 54(12):4095–4101. 16756331 10.1021/jf060536p

[23] Wen M, Han Z, Cui Y, Ho CT, Wan X, Zhang L. Identification of 4-*O*-p-coumaroylquinic acid as astringent compound of Keemun black tea by efficient integrated approaches of mass spectrometry, turbidity analysis and sensory evaluation. Food Chem. 2022;368:130803. 34403995 10.1016/j.foodchem.2021.130803

[24] Rösch D, Krumbein A, Mügge C, Kroh LW. Structural investigations of flavonol glycosides from sea buckthorn (*Hippophaë rhamnoides*) pomace by NMR spectroscopy and HPLC-ESI-MS n. J Agric Food Chem. 2004;52(13):4039–4046. 15212446 10.1021/jf0306791

[25] Marengo A, Fumagalli M, Sanna C, Maxia A, Piazza S, Cagliero C, Rubiolo P, Sangiovanni E, Dell’Agli M. The hydro-alcoholic extracts of Sardinian wild thistles (*Onopordum* spp.) inhibit TNFα-induced IL-8 secretion and NF-κB pathway in human gastric epithelial AGS cells. J Ethnopharmacol. 2018;210:469–476. 28916191 10.1016/j.jep.2017.09.008

[26] Marengo A, Maxia A, Sanna C, Bertea CM, Bicchi C, Ballero M, Cagliero C, Rubiolo P. Characterization of four wild edible Carduus species from the Mediterranean region via phytochemical and biomolecular analyses. Food Res Int. 2017;100(Pt 1):822–831. 28873755 10.1016/j.foodres.2017.07.071

[27] Xu D, Wang Q, Zhang W, Hu B, Zhou L, Zeng X, Sun Y. Inhibitory activities of caffeoylquinic acid derivatives from *Ilex kudingcha* C.J. Tseng on α-Glucosidase from *Saccharomyces cerevisiae*. J Agric Food Chem. 2015;63(14):3694–3703. 25805337 10.1021/acs.jafc.5b00420

