# Supplementary material for: Promising inhibition of diabetes-related enzymes and antioxidant properties of Ptilostemon casabonae leaves extract
Source: J Enzyme Inhib Med Chem. 2023 Oct 31;38(1):2274798. doi: 10.1080/14756366.2023.2274798 (PMC11003480; doi:10.1080/14756366.2023.2274798)
Supplement: Supplemental Material [file IENZ_A_2274798_SM4886.pdf]

**Supplementary Materials**

# Promising inhibition of diabetes-related enzymes and antioxidant properties of *Ptilostemon casabonae* leaves extract

Cinzia Sanna[a], Antonella Fais[b], Benedetta Era[b], Giovanna L. Delogu[b], Enrico Sanna[b], Laura Dazzi[b], Antonella Rosa[c], Arianna Marengo[d], Patrizia Rubiolo[d], Antonio De Agostini[a], Sonia Floris[b*#] and Francesca Pintus[b*#]

[a]*Department of Life and Environmental Sciences, University of Cagliari, via S. Ignazio da Laconi 13, 09123 Cagliari, Italy;*

[b]*Department of Life and Environmental Sciences, University of Cagliari, SS 554 – bivio per Sestu, 09042 Monserrato, Cagliari, Italy;*

[c]*Department of Biomedical Science, University of Cagliari, SS 554 – bivio per Sestu, 09042 Monserrato, Cagliari, Italy;*

[d]*Department of Drug Science and Technology, University of Turin, Via Pietro Giuria 9, 10125 Turin, Italy.*

*Correspondence: s.floris@unica.it, fpintus@unica.it

#These authors contributed equally to this work.

**Figure S1.** Lineweaver-Burk secondary plot of α-glucosidase inhibition by single compounds. **A**) Inverse plot of 1 ⁄ Vmax versus 1 ⁄ [1,5,Dicaffeoylquinic acid] for the $K_{IS}$ calculation; **B**) Inverse plot of 1 ⁄ Vmax (left) or slope (right) versus 1 ⁄ [apigenin] for the $K_{IS}$ and $K_I$ calculation; **C**) Inverse plot of 1 ⁄ Vmax (left) or slope (right) versus 1 ⁄ [rutin] for the $K_{IS}$ and $K_I$ calculation.

**A**

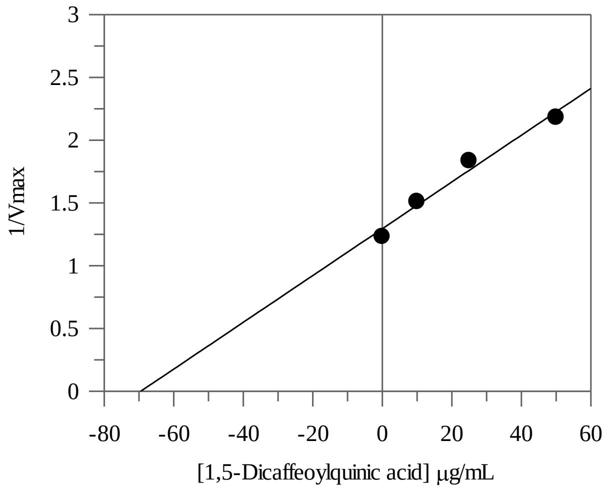


**B**

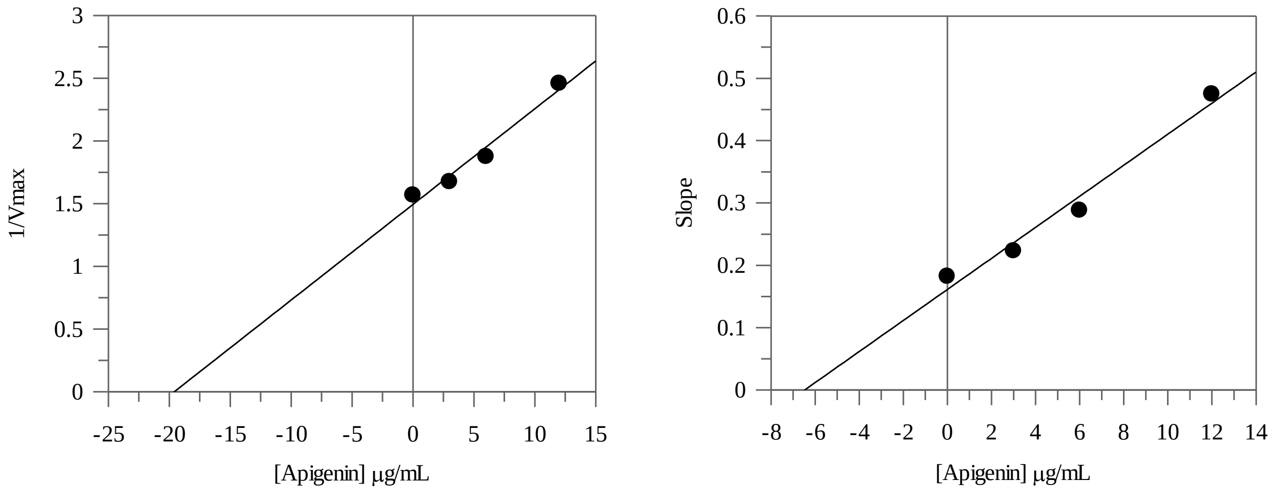

C

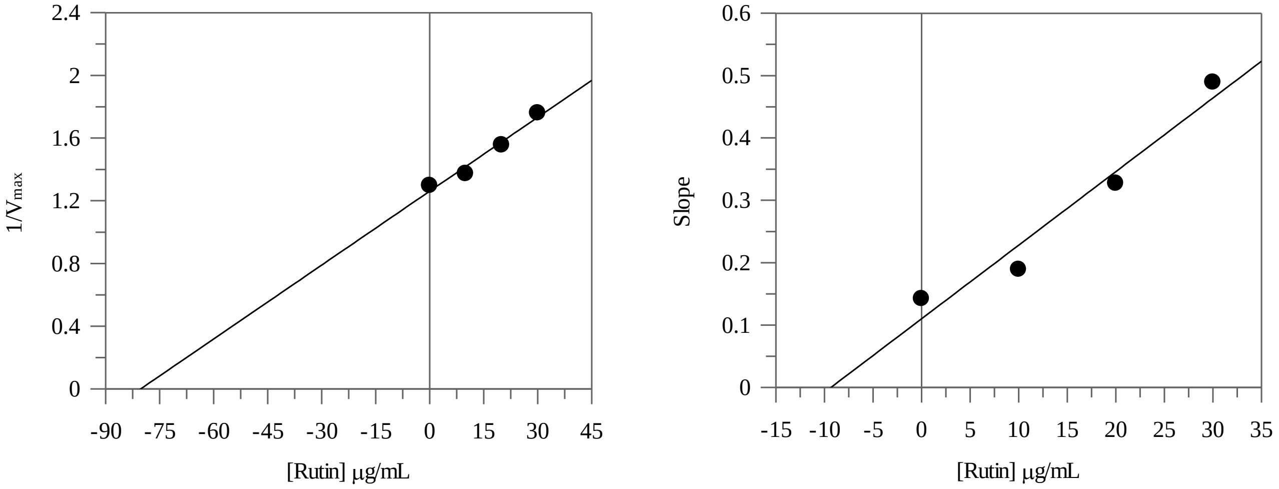
2.4
2
1.6
1.2
0.8
0.4
0
$1/V_{max}$
-90 -75 -60 -45 -30 -15 0 15 30 45
[Rutin] μg/mL
0.6
0.5
0.4
0.3
0.2
0.1
0
Slope
-15 -10 -5 0 5 10 15 20 25 30 35
[Rutin] μg/mL
